# Non-severe thermal burn injuries induce long-lasting downregulation of gene expression in cortical excitatory neurons and microglia

**DOI:** 10.3389/fnmol.2024.1368905

**Published:** 2024-02-27

**Authors:** Rebecca C. S. Ong, Jamie L. Beros, Kathy Fuller, Fiona M. Wood, Phillip E. Melton, Jennifer Rodger, Mark W. Fear, Lucy Barrett, Andrew W. Stevenson, Alexander D. Tang

**Affiliations:** ^1^Experimental and Regenerative Neuroscience, The University of Western Australia, Crawley, WA, Australia; ^2^Perron Institute for Neurological and Translational Sciences, Nedlands, WA, Australia; ^3^School of Biomedical Sciences, The University of Western Australia, Crawley, WA, Australia; ^4^Burn Injury Research Unit, The University of Western Australia, Crawley, WA, Australia; ^5^Burns Service of Western Australia, WA Department of Health, Murdoch, WA, Australia; ^6^Paediatric Burn Care, Telethon Kids Institute, Nedlands, WA, Australia; ^7^Menzies Institute for Medical Research, University of Tasmania, Hobart, TAS, Australia; ^8^School of Global and Population Health, The University of Western Australia, Crawley, WA, Australia

**Keywords:** burn injury, cortical neurons, microglia, astrocytes, transcriptome

## Abstract

Burn injuries are devastating traumas, often leading to life-long consequences that extend beyond the observable burn scar. In the context of the nervous system, burn injury patients commonly develop chronic neurological disorders and have been suggested to have impaired motor cortex function, but the long-lasting impact on neurons and glia in the brain is unknown. Using a mouse model of non-severe burn injury, excitatory and inhibitory neurons in the primary motor cortex were labelled with fluorescent proteins using adeno-associated viruses (AAVs). A total of 5 weeks following the burn injury, virus labelled excitatory and inhibitory neurons were isolated using fluorescence-activated cell sorting (FACS). In addition, microglia and astrocytes from the remaining cortical tissue caudal to the motor cortex were immunolabelled and isolated with FACS. Whole transcriptome RNA-sequencing was used to identify any long-lasting changes to gene expression in the different cell types. RNA-seq analysis showed changes to the expression of a small number of genes with known functions in excitatory neurons and microglia, but not in inhibitory neurons or astrocytes. Specifically, genes related to GABA-A receptors in excitatory neurons and several cellular functions in microglia were found to be downregulated in burn injured mice. These findings suggest that non-severe burn injuries lead to long lasting transcriptomic changes in the brain, but only in specific cell types. Our findings provide a broad overview of the long-lasting impact of burn injuries on the central nervous system which may help identify potential therapeutic targets to prevent neurological dysfunction in burn patients.

## 1 Introduction

Burn injuries are a form of trauma that can negatively affect an individual’s quality of life. Although burn injuries are commonly associated with chronic scarring and cellular changes at the injury site (e.g., the skin or lungs), organ systems remote from the injury site are also susceptible to long-lasting changes (Duke et al., 2017, 2019). The central nervous system (CNS) in particular, is known to have long-lasting dysfunction following a burn injury (Vetrichevvel et al., 2016). Previous epidemiological data has shown greater hospital readmissions and stay durations related to nervous system conditions (e.g., seizures and migraines) in burns patients with burns ranging from severe [≥20% total body surface area (TBSA)] to non-severe (<20% TBSA) compared to uninjured controls (Vetrichevvel et al., 2016). However, despite the prevalence and severity of the chronic CNS changes that develop in burn injury patients, little is known about the specific cellular functions and cell types within the brain that are affected.

Within the brain, neural circuits are composed of both neuronal and glial subtypes, with astrocytes and microglia having important roles in regulating neuronal function and immune responses (Vallejo et al., 2010). At the neuronal level, small cohort studies have provided clinical evidence for abnormal function in the cortex following a burn injury (Garside et al., 2018). Specifically, subsets of patients from small cohort studies have shown impaired neuronal excitability and plasticity in the motor cortex that persists for several years following a non-severe burn injury (Garside et al., 2018). Although there is limited knowledge regarding the effect of burn injuries on glial cells, peripheral nerve injuries which share several features of burn injuries whereby both involve damages to nerves outside of the CNS, are known to induce long lasting changes to both neurons and glia (Alvarado et al., 2013). Therefore, to better understand the mechanisms underlying neural dysfunction in the motor cortex, and gain insight to changes to cortical glial cells in general, characterising changes to the different neuronal and glial subtypes is needed. Here we use whole transcriptome sequencing as it provides a comprehensive overview of the possible functions and pathways that are altered by characterising the expression dynamics of genes (Ozsolak and Milos, 2011). Furthermore, transcriptomics has previously been used to identify the upregulation of genes associated with neuronal dysfunction and inflammation in dorsal root ganglion neurons (i.e., peripheral nervous system) in a mouse model of burn injury (Yin et al., 2016).

## 2 Materials and methods

### 2.1 Animals

For this study, a comparison between burn-injury and sham injury groups was made, with the gene expression data from each mouse representing the experimental unit.

All animal procedures were performed in accordance with the Australian code of practice for the care and use of animals for scientific purposes and were approved by the University of Western Australia Animal Ethics Committee (2021/ET00021). All authors complied with the ARRIVE guidelines. Female C57BL/6J mice, at 12-weeks old were pair-housed in a temperature-controlled facility (24°C) under a standard 12-h light/dark cycle with *ad libitum* access to food and water. A total of 24 mice were used in this study. Upon arrival, mice were randomly assigned to a burn injury (*n* = 11) or sham treatment (*n* = 13) group and were given 7 days to acclimatise to the research facility before experimentation.

### 2.2 Stereotaxic injections

A total of 3 days prior to stereotaxic surgery, paracetamol was dissolved into the drinking water (1 mg/ml) to acclimatise the animals to the analgesic. Stereotaxic surgery was performed on all mice in preparation for gene delivery of adeno-associated viruses (AAVs) that express fluorophores selectively in excitatory or inhibitory neurons. Mice were administered prophylactic analgesia at least 30 min prior to surgery (0.1 mg/kg buprenorphine, sub-cutaneous). Isoflurane inhalation (5% induction with 1 L oxygen/min, 0.5–2.5% maintenance with 1 L oxygen/min) was used to deeply anaesthetize the animal, as verified by an absence of the hind paw withdrawal reflex. Fur on the scalp was trimmed and disinfected (5% chlorhexidine followed by 0.5% chlorhexidine in 70% alcohol) prior to placing the animal in a stereotaxic frame. A line block of local anaesthetic (0.1 ml of 2.5 mg/ml bupivacaine, sub-cutaneous) was injected along the midline of the shaved area before a 12–15 mm incision into the scalp was made, exposing the skull. Throughout surgery, animals were kept hydrated (10 ml/kg/h compound sodium lactate (Hartmann’s) solution administered in doses every 15 min) and monitored for breathing rate and temperature. Animals were kept on a heating pad for the duration of surgery to maintain body temperature.

Using a high-speed drill, craniotomies (<1 mm in diameter) were made over the left and right primary motor cortices (M1 coordinates 0.5 mm anterior and 1.5 mm lateral from bregma). A glass micropipette connected to a Nanoliter injector (World Precision Instruments Sarasota, USA) was used to deliver viral injections into the M1 region of both hemispheres. Bilateral injections of an AAV mixture, containing a 1:1 ratio of pENN.AAV.CamKII0.4.eGFP.WPRE.rBG (AAV5-CamKII-eGFP, Addgene, #105541-AAV5) and pAAV-mDlx-NLS-mRuby2 (AAV1-mDlx-mRuby2, Addgene, #99130-AAV1), was used. This was to express enhanced green fluorescent protein (eGFP) in neurons positive for the CaMKII promoter, commonly used to target excitatory neurons (Nathanson et al., 2009; Mukherjee et al., 2020; Ibrahim et al., 2024). The expression of mRuby2 in neurons positive for the mDlx enhancer, was done to target inhibitory neurons (Dimidschstein et al., 2016; Hoshino et al., 2021; Kaur and Berg, 2022). To maximise coverage of all cortical layers, one injection (200 nl per injection at a rate of 40 nl/min) was made at the depths of 200 and 450 μm from the brain surface. Following the administration of each injection, the micropipette was left undisturbed for 1 min before being raised. The incision site was sutured closed, and a local anaesthetic (0.1 ml of 2.5 mg/ml bupivacaine) was administered topically to the sutures.

### 2.3 Burn injury

Whilst under anaesthesia, the right flank of the animal was shaved (∼20 mm diameter) immediately following virus injection surgery. Mice in the burn injury group received a full thickness burn injury (19 mm in diameter) by placing a solid brass cylinder (65 g, 19 mm in diameter) heated to 95°C against the shaved skin. This equated to a burn approximating 7–8% of the animal’s total body surface area (TBSA), reflecting a non-severe burn model (Bruen et al., 2004). Sham treatment mice underwent all procedures with the exception of the burn injury (i.e., shaved but not burned). Mice were transferred to a heating pad immediately after surgery and monitored every 15 min until complete recovery. Post-surgical analgesia (0.05–0.1 mg/kg buprenorphine, sub-cutaneous) was administered on a case-by-case basis if the animal displayed signs of discomfort. Five burn-injured mice required 0.05 mg/kg buprenorphine post-surgery. Paracetamol-instilled (1 mg/ml) water was supplied to all animals for a further 5 days post-surgery.

### 2.4 Tissue dissociation and myelin removal

Mice were euthanised 5 weeks post-surgery using a two-step process of isoflurane anaesthesia (5% with 1 L oxygen/min) followed by an overdose of sodium pentobarbitone (160 mg/kg, intraperitoneal). Cardiac perfusion with 20 ml of ice-cold saline (0.9%) was performed prior to dissection of both motor cortical hemispheres. Cortical regions caudal to the motor cortices were also dissected to obtain an unstained (i.e., no virus labelled) control sample and for staining and sorting glial cells of interest. Dissected cortical tissues were immediately transferred into separate 2 ml tubes (motor cortex and caudal cortical regions) containing ice-cold Hank’s Balanced Salt Solution (HBSS) without calcium and magnesium (Sigma-Aldrich, #55021C). The weight of each tissue section was recorded prior to dissociation.

Neural tissues were dissociated to obtain a single-cell suspension using the Neural Tissue Dissociation Kit (P) (Miltenyi Biotec, #130-092-628), according to the manufacturer’s protocol. A subsequent myelin debris depletion was performed on the single-cell suspension, by magnetic activated cell sorting (MACS) using Myelin Removal Beads II (Miltenyi Biotec, #130-096-733) as per the manufacturer’s instructions.

### 2.5 Neuronal and glial cell isolation

Following myelin depletion, cells obtained from the motor cortex, and from caudal cortical regions were resuspended in 1 ml HBSS with calcium and magnesium [HBSS(w)] (Thermo Fisher, #14025076) containing RNasin ribonuclease inhibitors (1:200, Promega, #N2515) in preparation for antibody staining. Washing steps were performed at 300 × g at 4°C for 4 min. A proportion of cells obtained from cortical tissue caudal to the motor cortex were aliquoted into 5 ml Falcon^®^ tubes and used as FACS controls. Single-stain antibody controls included: live/dead Fixable Viability Dye eFluor780 (1:250, Thermo Fisher, #65-0865-14), CD11b-eFluor450 (1:160, eBioscience, #48-0112-82), and ACSA2-PE (1:50, Miltenyi Biotec, #130-116-244). The astrocyte cell surface antigen 2 (ACSA2) antibody has been well-characterised to target and isolate cortical astrocytes. The cluster differentiation molecule 11b (CD11b) antibody was used to isolate microglia. An unstained control was also included.

All the cells taken from the motor cortex, and the remaining cells from the cortical tissue caudal to the motor cortex were washed with HBSS(w) before being incubated with the live/dead Fixable Viability Dye eFluor780 for 20 min, in the dark on ice. Cells were then washed with three rounds of ice-cold FACS buffer [HBSS(w), 1% fetal bovine serum (FBS) and 2 mM ethylenediaminetetraacetic acid (EDTA)]. Following the third wash, motor cortex cells were resuspended in 200 μl FACS buffer [made with HBSS without calcium, magnesium, and phenol red (Thermo Fisher, #14175095), 1% FBS, 2 mM EDTA and 1:200 RNasin]. Cells from the cortical regions caudal to the motor cortex underwent further co-staining with ACSA2-PE and CD11b-eFluor450 for 15 min, in the dark on ice, followed by three rounds of FACS buffer wash. These cells were resuspended in 200 μl of the same FACS buffer used for motor cortex cells. All control samples were subjected to the same immunolabelling protocol, however, were pseudo-stained with HBSS(w) when a particular antibody wasn’t required.

Cell sorting was performed on a BD FACSAria™ III Cell Sorter using the FACSDiva v8.0.1 Software. Unstained and single-stained controls for the different virus and antibody labelled cells were used to determine the compensation parameters and gating strategy. Cells obtained from the motor cortex, and cells obtained from the cortical regions caudal to the motor cortex were sorted separately using the appropriate laser lines to isolate each of the four cell populations: violet 405 nm, blue 488 nm, and yellow-green 561 nm. Samples were run through a 100 μm sort nozzle at 20 psi and single cells were directly sorted into lysis Buffer RLT (Qiagen, #74004).

### 2.6 Imaging flow cytometry

Imaging flow cytometry was performed on an Amnis^®^ ImageStream^®^ X Mk II (Luminex Corp), with INSPIRE acquisition software (v4.1). Excitation lasers 200 mW 405 nm (CD11b-e450), 200 mW 488 nm (CamKII-eGFP), 200 mW 561 nm (mDlx-mRuby2, ACSA2-PE) and 150 mW 652 nm (Viability) were used for analysis. A 1.5 mW 785 nm laser was used to provide a side scatter signal and measurement of SpeedBeads (Amnis) for internal calibration. Single-stained AbC™ Total Antibody Compensation Beads using FITC, and PE were used to calculate a compensation matrix for eGFP and mRuby. All images were captured at 60x magnification. Cells were identified in a scatter plot of the brightfield (BF1) aspect ratio vs. area. Analysis of acquired imaging flow cytometry data was performed using IDEAS image analysis software (v6.3).

### 2.7 RNA extraction and sequencing

RNA from sorted neuron and glia samples was isolated using the RNeasy Micro Kit (QIAGEN, Cat. 74004) as per manufacturer’s instructions. All RNA samples were externally quantified using the 2100 Bioanalyzer (Agilent Technologies). RNA integrity number (RIN) and concentration was evaluated for each sample, with a requirement for sequencing of a RIN > 5 and at least 1 ng total RNA. Samples that didn’t meet the requirements were excluded (see Supplementary Table 1).

RNA samples (*n* = 63) were sent to the Australian Genome Research Facility (AGRF, Melbourne, VIC, Australia) where RNA-seq was performed. In summary, ribosomal RNAs (rRNA) were depleted from samples using the Illumina Ribo-Zero Plus rRNA Depletion protocol prior to the formation of cDNA libraries using the Illumina Stranded Total RNA Library Preparation Kit. Whole transcriptome sequencing was performed on the Illumina NovaSeq 6000, generating 50 million, 150 base pair (bp) paired-end reads using the Illumina bcl2fastq 2.20.0.422 pipeline.

### 2.8 RNA-seq analysis

The quality of returned raw RNA-seq data was checked using FastQC (v0.11.3). The first 15 bp of reads were trimmed using *fastp* (Chen et al., 2018) and sequence quality evaluation of the cleaned reads showed an average Q30 base rate of 91.33% (the lowest value at 89.50% and the highest value at 93.39%) indicating high sequencing accuracy (see Supplementary Table 1). Reads were aligned to the Ensembl *Mus musculus* mm10 reference genome (version GRCm39) using the STAR RNA-seq aligner (v2.7.1) (Dobin et al., 2013). The average unique mapping rate of transcripts across all samples was 61.93% (Supplementary Table 1). STAR-aligned SAM files were converted into BAM files, using SAMtools (v1.11) (Li et al., 2009), for more efficient downstream processing. In R, mapped reads were counted using GenomicAlignments (v1.26.0). Using DESeq2 (v1.30.1) (Love et al., 2014), gene counts were normalised and transformed using the variance stabilizing transformations (VST) function to account for library size differences between samples. Visualisation of raw and transformed counts was performed to assess the quality of data across samples. Raw and VST transformed count data for sample number 8 in inhibitory neurons was identified as an outlier and thus was excluded from further analyses (Supplementary Figure 1).

Differential expression analysis was subsequently performed using the *DESeq* function in DESeq2. Correction for batch sequencing effects was also implemented in the analysis. *P-*values of differentially expressed genes (DEGs) were adjusted by the Benjamini-Hochberg false discovery rate correction (p.adj) for multiple comparisons. Significant DEGs were selected based on an absolute log_2_ fold-change (log2FC) ≥1 and p.adj ≤ 0.05.

## 3 Results

### 3.1 Fluorescent activated cell sorting (FACS) virus labelled neurons isolated a population of neurons only positive for CaMKII and a subset of neurons double-positive for CaMKII and mDlx

**FIGURE 1:**
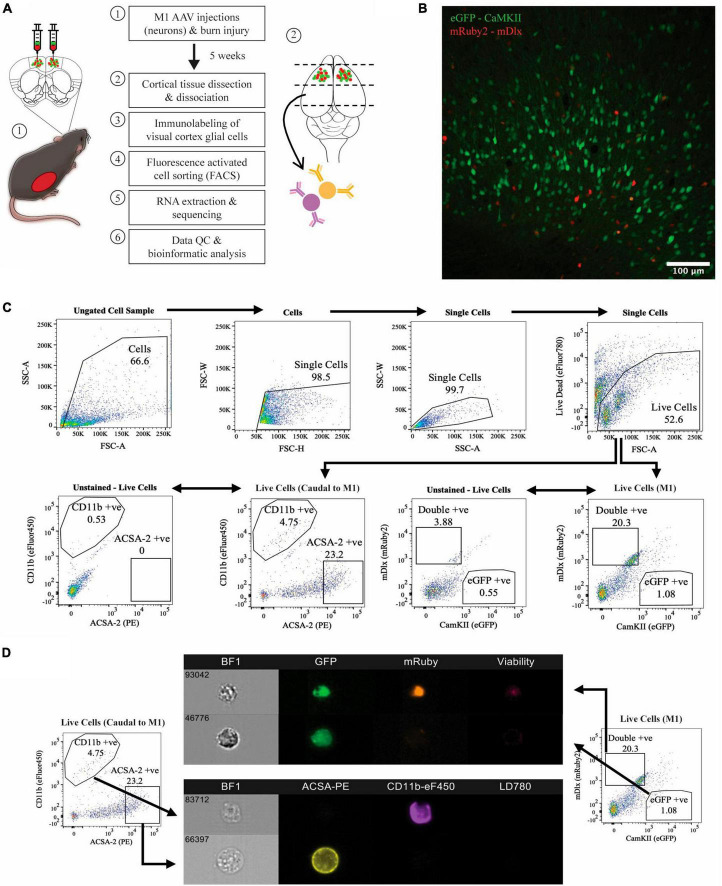
Isolation of four major cell populations from mouse cortical tissue. **(A)** Schematic of the experimental protocol. **(B)** Histology used to confirm AAV transduction of neurons, captured at 20x magnification using confocal microscopy. **(C)** Hierarchial gating strategy used to obtain live eGFP-positive neurons and mRuby2-positive neurons from M1 tissue of all mice. ACSA-2 positive astrocytes and CD11b positive microglia were obtained from cortical tissue caudal to the motor cortex of all mice, employing the same gating strategy. Plots generated from <50,000 events. Gated population numbers indicate percentage of total events displayed in each pseudocolour density plot. **(D)** Sorted cell populations were visualised on six-channels using an Amins^®^ imaging flow cytometer. BF1, brightfield.

To investigate the effects of thermal burns on different cortical cell types, we used FACS to isolate virus-labelled neuronal subtypes from the motor cortex and antibody-tagged glial populations in cortical regions caudal to the motor cortex in burn and sham-injured mice (Figure 1A). As expected, histological examination of the motor cortex in coronal brain sections showed that the majority of the AAV transduced neurons were positive for CaMKII (i.e., expressing eGFP), suggesting a greater proportion of excitatory neurons present compared to inhibitory (i.e., expressing mRuby2 or eGFP and mRuby2) (Figure 1B). Surprisingly, no single-labelled mRuby2-positive cell population was detected during FACS acquisition plot but there was a defined cluster of cells expressing both eGFP and mRuby2 fluorescence (i.e., double-positive) was identified. To rule out the possibility that the double labelling observed during FACS was a by-product of sheared neuronal processes expressing eGFP adhering to mRuby2 inhibitory cells, or *vice versa*, imaging flow cytometry was performed (Figure 1D). Upon imaging, live, intact eGFP-positive mRuby2-negative cells were verified, confirming the isolation of virus labelled CaMKII only positive neurons. Similar structural properties were identified when imaging the double-positive cell population (i.e., live and intact cells), however, the eGFP and mRuby2 fluorophore signals were found to be co-localised within the cell body. Thus, the sorted double-positive population was categorised as the subset of inhibitory neurons known to express CaMKII (Keaveney et al., 2020). Astrocytes and microglia were immunolabelled and FACS isolated from cortical tissue obtained caudal to the motor cortex. FACS acquisition plots identified two distinct cell populations defined by their high fluorescence for ACSA2 and CD11b (Figure 1C). A portion of isolated ACSA2-positive and CD11b-positive cells underwent flow cytometry and showed clear cellular morphology with distinct antibody labelling throughout the cell (Figure 1D).

### 3.2 Thermal burn injury alters the expression of *Gabrg2* in CaMKII positive neurons but not of any gene with known function in CaMKII-mDlx double-positive neurons 5 weeks post-injury in the motor cortex

**FIGURE 2:**
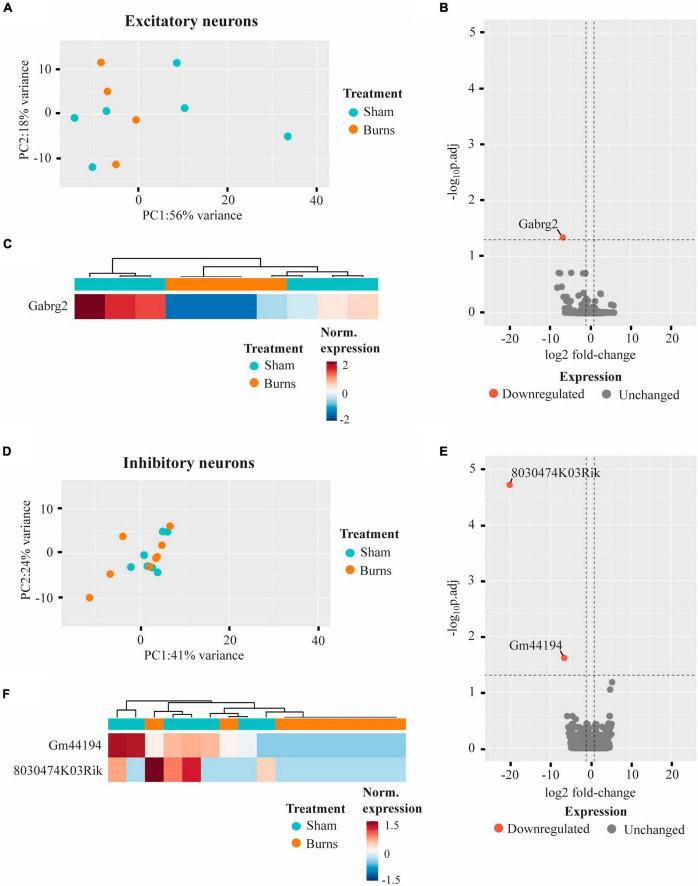
Gene expression profiles of motor cortex excitatory and inhibitory neurons are not significantly altered 5-weeks following a thermal burn injury. **(A)** PCA of FACS-purified excitatory neurons from burn- (*n* = 4) and sham- (*n* = 6) injured mice reveal large heterogeneity between samples. **(B)** Volcano plot and **(C)** heatmap display only one gene that was differentially regulated in excitatory neurons after a burn. **(D)** PCA of FACS-purified inhibitory neurons from burn- (*n* = 9) and sham- (*n* = 7) display no clear clustering of sample groups. **(E)** Volcano plot and **(F)** heatmap display two differentially expressed genes in inhibitory neurons after a burn. See also Supplementary Tables 2, 3.

Whole transcriptome bulk RNA-sequencing of sorted only CaMKII positive neurons revealed a large degree of within-group heterogeneity, with no clear clustering of burn and sham-injured samples when visualised on a principal component analysis (PCA) plot (Figure 2A). Reflecting this, differential expression analysis revealed only one gene that was significantly downregulated in CaMKII positive neurons (*Gabrg2*, log2FC = −6.74) 5-weeks following a burn injury (Figures 2B, C and Table 1). Significant genes were identified based on an absolute log_2_ fold-change (log2FC) ≥1 and adjusted *p-*value for multiple comparisons (p.adj) ≤ 0.05. A table of raw read counts for all significant differentially expressed genes identified was also extracted to examine the expression of each gene across individual burn and sham samples (Supplementary Tables 2–5). For CaMKII-mDlx double-positive neurons, a PCA plot revealed little separation between the transcriptomic profiles of burn and sham injured mice (Figure 2D). Differential expression analysis revealed two genes, in CaMKII-mDlx double-positive neurons, that had a significant change in expression (*Gm44194*, log2FC = −6.51; *8030474K03Rik*, log2FC = −20.10) 5-weeks post-burn injury compared to sham-injured controls (Figures 2E, F and Table 1). Genes annotated with “*Gm-*” or “*Rik”* represent long non-coding RNAs, antisense transcripts or coding RNAs of unknown origin that have not yet been assigned a canonical name. Due to the low number of significant differential genes identified, no gene ontology (GO) classifications could be generated for either CaMKII positive or CaMKII-mDlx double-positive neurons.

**TABLE 1:** Summary of genes that displayed a significant differential expression in neurons and glial cells of the mouse cortex 5-weeks following a non-severe burn injury.

	Symbol	Gene name	log2 FC	p.adj
Excitatory neurons	Gabrg2	Gamma-aminobutyric acid (GABA) A receptor, subunit gamma2	−6.74	0.05
Inhibitory neurons	Gm44194	Predicted gene, 44194	−6.51	2.44e-02
	8030474K03Rik	RIKEN cDNA 8030474K03 gene	−20.10	1.90e-05
Astrocytes	Gm29326	Predicted gene, 29326	6.58	0.01
	Gm26493	Predicted gene, 26493	5.72	0.02
	Gm41639	Predicted gene, 41639	5.36	0.01
	Gm10722	Predicted gene, 10722	4.41	0.02
	Gm37415	Predicted gene, 37415	−5.96	0.05
Microglia	Shank3	SH3 and multiple ankyrin repeat domains 3	−1.77	0.01
	Ier5l	Immediate early response 5-like	−1.89	0.03
	Grb14	Growth factor receptor bound protein 14	−6.61	0.03

Significant genes were selected based on a log_2_ fold change (log2 FC) > 1 and an adjusted *p*-value (p.adj) ≤0.05.

### 3.3 Thermal burn injury alters the expression of a small number of genes in CD11b-positive microglia but not of any genes with known function in ACSA2-positive astrocytes 5 weeks post-injury

**FIGURE 3:**
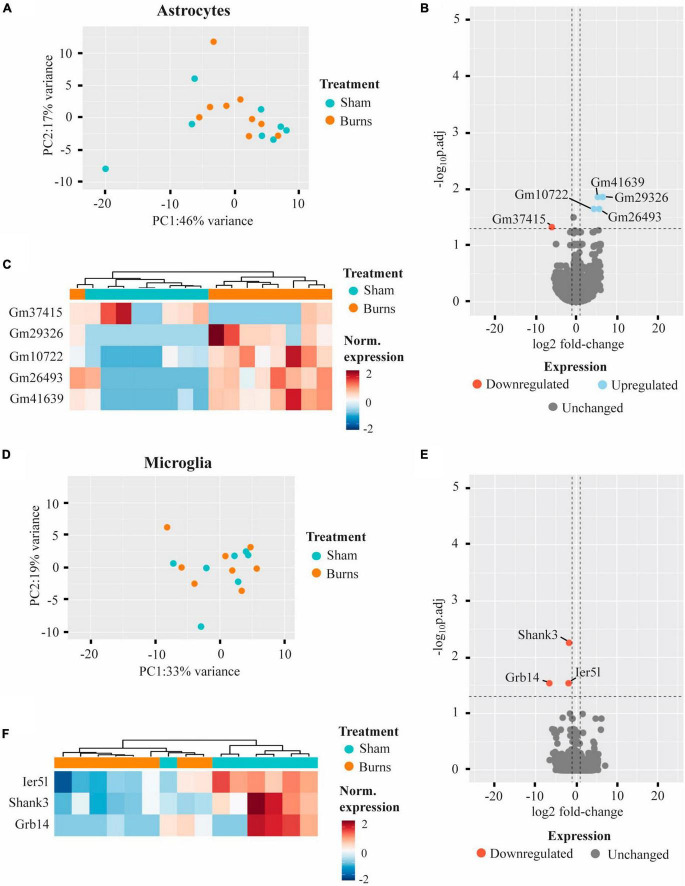
Burn injuries have little effect on the gene expression profiles of astrocytes and microglia 5-weeks following a thermal burn injury. **(A)** PCA of FACS-purified astrocytes from burn- (*n* = 9) and sham- (*n* = 8) injured mice reveal no clear difference between groups. **(B)** Volcano plot and **(C)** heatmap display several genes differentially regulated in astrocytes after a burn. **(D)** PCA of FACS-purified microglia from burn- (*n* = 8) and sham- (*n* = 7) display no clear clustering of sample groups. **(E)** Volcano plot and **(F)** heatmap display few differentially expressed genes in microglia after a burn. See also Supplementary Tables 4, 5.

Comparing the transcriptomic profiles of ACSA2-positive astrocytes obtained from burn and sham injured mice 5-weeks post-burn, a PCA plot indicated that a burn injury had little effect on gene expression, with biological replicates from each group clustering in close proximity (Figure 3A). Indeed, differential expression analysis revealed five genes that had an altered expression in ACSA2-positive astrocytes (four upregulated, one downregulated) following a burn injury (Figures 3B, C and Table 1). However, all the genes identified began with the annotation “*Gm-*” and thus, their functional significance could not be identified. Similarly, a PCA plot of CD11b-positive microglia from burn and sham injured mice showed no distinct separation between treatment groups, reflecting no strong effect of burn injuries on microglial gene expression (Figure 3D). Nonetheless, differential expression analysis revealed three genes (*Shank3*, FC = −1.77; *Ier5l*, FC = −1.89; *Grb14*, FC = −6.61) that all displayed a decreased expression in CD11b-positive microglial cells post-burn (Figures 3E, F and Table 1).

## 4 Discussion

Given that burn injury patients have been shown to have long-lasting neuronal excitability and plasticity impairments in the motor cortex, we sought to characterise the long-lasting transcriptomic changes to excitatory and inhibitory neurons in the primary motor cortex, as well as microglia and astrocytes in general 5-weeks following a thermal burn injury. Surprisingly, burn injury only had a long-lasting effect on a small number differentially expressed genes identified which was cell-type specific. In both CaMKII-mDlx double-positive inhibitory neurons from the motor cortex and cortical ACSA2-positive astrocytes caudal to the motor cortex, only genes related to long-coding RNAs, antisense transcripts or coding RNAs were identified. However, in CaMKII-positive excitatory neurons and CD11b-positive microglia, genes related to GABA receptor function (*Gabrg2*), synaptic plasticity and the immune response were found to be significantly down regulated (*Shank3*, *ler5l*, and *Grb14*).

Our study focussed on the neuronal changes in the motor cortex as this region has previously been implicated by clinical studies to have abnormal neuronal function (Garside et al., 2018) and plasticity (Whife et al., 2020) following a thermal burn injury. From the significant DEGs, identifying impaired neuronal functions and processes was difficult as the majority of differentially expressed genes identified were functionally uncharacterised and gene set pathway enrichment analysis was therefore not possible. However, it is known that *Gabrg2* encodes for the γ2 subunit on Gamma-aminobutyric acid-A (GABA_A_) receptors, the subunit critical for clustering GABA_A_ receptors to the post-synapse (Alldred et al., 2005). Given the large effect size of the downregulation (log_2_FC > 6), our result suggests impaired inhibition of CaMKII-positive excitatory neurons in the motor cortex, potentially underlying a pathological excitatory state of the motor cortex. This result partially supports the reduced inhibitory function (cortical silent period) observed in the motor cortex from a small number of burn injury patients with <10% TBSA injuries (Garside et al., 2018). However, it is worth noting that reduced inhibitory function was not observed in all burn injury patients studied and only occurred in specific subsets. Furthermore, the cortical silent period induced with transcranial magnetic stimulation (TMS) is a non-specific measure of motor cortex function and is assumed to involve both cortical and spinal circuits, and GABA_A_ and GABA_B_ receptor activity (Ziemann et al., 2015). Nevertheless, the downregulation of *Gabrg2* in excitatory neurons in the motor cortex may have significant clinical relevance in addition to altered motor control, such as mental health conditions where loss/absence of *Gabrg2* has been associated with anxiety behaviuors in *Gabrg2* knockout mice (Chandra et al., 2005).

In cortical CD11b-positive microglia caudal to the motor cortex, we found a significant down regulation of *Ier5*l, *Shank3*, and *Grb14*. To date very little is known of the function of the immediate early response gene *Ier5l* beyond having a role in transcriptional regulation (Ueda et al., 2019) and cellular development (Li et al., 2014). In contrast, *Shank3* codes for a scaffolding protein primarily localised post-synaptically at excitatory synapses (Uchino et al., 2006) and is thought to be a regulator of long-term potentiation. The role of *Shank3* has not been well characterised in microglia but is known to be chronically up regulated in expression following a traumatic brain injury (Makinde et al., 2020) and leads to a decreased gliosis response in *Shank3* knockout mice (Urrutia-Ruiz et al., 2022). Similarly, the role of *Grb14* in microglia has not been characterised, but its protein form is a known regulator of insulin and insulin-like growth factor signalling pathways (Holt and Siddle, 2005), that may play a role in mediating the inflammatory response of microglia (Inglis et al., 2012; Brabazon et al., 2018). Based on these previous findings, our results suggest that elements of the synaptic and gliosis functions of cortical microglia are down-regulated 5 weeks post-injury, but future validation is required to confirm this.

Comparing our results in the cortex to studies in the peripheral nervous system suggests that neural cells in the periphery are more susceptible to long-lasting impairment. For example, in rats that received a full-thickness 20% TBSA burn injury, decreases in the conduction velocities of peripheral sensory and motor nerves were observed up to 4-weeks post-burn (Higashimori et al., 2005). Similarly, 35% TBSA burn injuries in mice have been shown to result in increased microglial densities, and microglia-associated inflammatory markers surrounding motor neurons 2-weeks post-burn (Ma et al., 2019). These lasting microglial changes have also been seen with non-severe burn injuries (i.e., less than 20% TBSA in adults) (Fear et al., 2017). In rodent models of 3–4% TBSA burn injuries, reductions in nociceptive sensitivity at the burn wound were found to remain for up to 8-weeks post-burn, coupled with decreases in nerve densities at sites close and remote to the wound (Morellini et al., 2012; Palanivelu et al., 2018). Notably, non-severe burns in mice also increase the activation of spinal astrocytes and microglia when measured 2-weeks post-burn (Shields et al., 2012). While the differences in observation timepoint and burn injury model, such as the location and TBSA of the burn prevent direct comparison to our study, burn-induced changes to the peripheral nervous system seem more prominent than the CNS.

While our study has identified specific changes to gene expression in the cortex following a peripheral burn injury, future work is needed to identify *how* these chronic changes occur. Previous studies in rodents have shown that peripheral burn injuries increase the permeability of the blood brain barrier (Patel et al., 2008; Yang et al., 2020), which presumably provides a pathway for burn-induced inflammatory mediators in the periphery to interact with neurons and glia in the brain. In addition, burn injuries are known to affect efferent (Patwa et al., 2019) and afferent (Chang et al., 2010) neurons in the spinal cord, possibly leading to altered signalling and or connectivity with the cortex, however, a direct link between burn-induced changes in the spinal cord and brain has yet to be demonstrated.

To increase the specificity of our transcriptomic characterisation we used viral labelling with commercially available vectors for specific promoters to separate excitatory and inhibitory neurons. Using the CaMKII-eGFP AAV we primarily targetted cortical pyramidal neurons, as it has been shown that CaMKII expressing cells are predominantly excitatory (Nathanson et al., 2009). However, it is known that the CaMKII promoter also results in some labelling of inhibitory neurons (Keaveney et al., 2020; Veres et al., 2023). Therefore, by combining our CaMKII-eGFP AAV with the mDlx-mRuby2 AAV to target inhibitory neurons, we were able to separate the two cell types through FACS to some extent. Specifically, we interpreted cells that were eGFP-positive and mRuby2-negative to be excitatory neurons, whereas cells double-positive for eGFP and mRuby2 (i.e., expressing both CaMKII and mDlx) to be inhibitory neurons. To precisely separate the two cell types, future studies should consider the use of a single bicistronic vector containing two separate cassettes with either the CaMKII or mDlx promoter. However, to our knowledge, such a vector has not been used and is not available commercially. In addition, it is important to note that we used the expression CD11b as our sole marker to identify and isolate cortical microglia cells. While CD11b is a widely used microglia marker, it is also known to be found in several other cells from the myeloid lineage (Dziennis et al., 1995) (e.g., macrophages). Through our use of cardiac saline perfusion to remove perivascular immune cell types, we are confident that our isolated cells are CD11b-positive CNS microglia (Nikodemova and Watters, 2012; Bordt et al., 2020; Rayaprolu et al., 2020), but cannot exclude the possibility that our also contains some infiltrating macrophages and other CD11b-positive cells.

In addition to our viral strategy, we implemented an extra “sanity check” with imaging flow cytometry for our neuron and glial cell types, which validated that our sorted cells were whole single cells expressing the correct fluorophores. We suggest that future studies also integrate imaging flow cytometry into their workflow where possible as it provides a valuable control following cell sorting, particularly when using promoter-based gene targetting strategies that could label multiple cell types.

In conclusion, our results provide further evidence that burn injuries lead to long lasting effects on the primary motor cortex in a cell specific manner. Furthermore, we provide the first characterisation of the long-lasting transcriptomics changes to glial cells in the CNS following a burn injury and show that microglia in particular show long-lasting transcriptomic changes. Further characterisation of neurons and glia outside the motor cortex, including non-cortical regions (e.g., the cerebellum) is needed to fully understand the effect of burn injuries on the brain. However, our data provides a foundation to identify therapeutic targets for the prevention of chronic neurological disorders in burn injury patients.

## Data availability statement

The data present in the study are deposited in the Gene Expression Omnibus (GEO) repository, accession number GSE229384 and can be found here: https://www.ncbi.nlm.nih.gov/geo/query/acc.cgi?acc=GSE229384.

## Ethics statement

The animal study was approved by the University of Western Australia Animal Ethics Committee (2021/ET00021). The study was conducted in accordance with the local legislation and institutional requirements.

## Author contributions

RO: Conceptualization, Data curation, Formal analysis, Investigation, Methodology, Validation, Visualization, Writing – original draft, Writing – review and editing. JB: Investigation, Supervision, Writing – review and editing. KF: Methodology, Resources, Visualization, Writing – review and editing. FW: Funding acquisition, Writing – review and editing. PM: Methodology, Writing – review and editing. JR: Funding acquisition, Writing – review and editing. MF: Conceptualization, Funding acquisition, Writing – review and editing. LB: Investigation, Writing – review and editing. AS: Conceptualization, Funding acquisition, Investigation, Supervision, Writing – review and editing. AT: Conceptualization, Funding acquisition, Investigation, Project administration, Supervision, Writing – original draft, Writing – review and editing.
