# Supplementary material for: Non-severe thermal burn injuries induce long-lasting downregulation of gene expression in cortical excitatory neurons and microglia
Source: Front Mol Neurosci. 2024 Feb 27;17:1368905. doi: 10.3389/fnmol.2024.1368905 (PMC10927825; doi:10.3389/fnmol.2024.1368905)
Supplement: Supplementary file 1 [file Data_Sheet_1.docx]

Supplementary Material

Non-severe thermal burn injuries induce long-lasting downregulation of gene expression in cortical excitatory neurons and microglia

Rebecca C S Ong*, Jamie L Beros, Kathy Fuller, Fiona M Wood, Phillip E Melton, Jennifer Rodger, Mark W Fear, Lucy Barrett, Andrew W Stevenson, Alexander D Tang

*** Correspondence:** Alexander D Tang: adtang.research@gmail.com

# Supplementary Figures and Tables

## Supplementary Figures

**B**

**A**

**Supplementary Figure 1. Bar plot of raw and variance stabilising transformed (VST) counts of all sequenced FACS-isolated cell types, related to STAR Methods. A)** Raw counts (i.e., library size) of sequenced excitatory neurons, inhibitory neurons, astrocytes and microglia samples taken from the cortex of burn and sham injured mice. **B)** Counts were transformed using the VST function account for library size differences. VST counts for sample number 8 in inhibitory neurons was noticeably different and was thus, removed from subsequent analyses.

**Table S2: Raw read counts of genes with a significant differential expression in excitatory neurons between burn and sham samples, related to Figure 2C.**

**Table S3: Raw read counts of genes with a significant differential expression in inhibitory neurons between burn and sham samples, related to Figure 2F.**

**Table S5: Raw read counts of genes with a significant differential expression in microglia between burn and sham samples, related to Figure 3F.**

**Table S4: Raw read counts of genes with a significant differential expression in astrocytes between burn and sham samples, related to Figure 3C.**
