# Supplementary material for: Non-severe thermal burn injuries induce long-lasting downregulation of gene expression in cortical excitatory neurons and microglia
Source: Front Mol Neurosci. 2024 Feb 27;17:1368905. doi: 10.3389/fnmol.2024.1368905 (PMC10927825; doi:10.3389/fnmol.2024.1368905)
Supplement: Supplementary file 2 [file Table_1.PDF]

Table S1: Details of sorted excitatory neurons, inhibitory neurons, astrocytes and microglial cells and RNA samples, obtained from animals used in the study, related to STAR Methods. Rows in bold indicate the final RNA samples from burn and sham-injured mice used in the differential gene expression analysis.

Animal ID	Batch #	Treatment	Cell type	FACS-sorted cell number	RNA (ng)	RIN	Total # raw reads	Q30 base rates	STAR Mapping rate	Notes
1	1	Sham	Excitatory neurons	–	–	–	–	–	–	Unexpected adverse event; early EOE
**2**	**1**	**Burns**	**Excitatory neurons**	**11,484**	**6.9**	**9.5**	**475,464,316**	**92.77%**	**59.82%**	
3	1	Burns	Excitatory neurons	5,527	7.3	9	125,060,132	92.99%	62.74%	Removed from analysis due to skin ulceration
4	1	Sham	Excitatory neurons	–	–	–	–	–	–	Unexpected adverse event; early EOE
5	1	Sham	Excitatory neurons	9,845	0.0	1	–	–	–	Insufficient RNA quantity for sequencing
6	1	Burns	Excitatory neurons	5,614	0.0	1	–	–	–	Insufficient RNA quantity for sequencing
7	1	Burns	Excitatory neurons	1,632	0.7	1	–	–	–	Insufficient RNA quantity for sequencing
**8**	**1**	**Sham**	**Excitatory neurons**	**2,257**	**1.0**	**9.7**	**99,575,249**	**92.04%**	**41.00%**	
9	1	Sham	Excitatory neurons	–	–	–	–	–	–	Unexpected adverse event; early EOE
10	1	Burns	Excitatory neurons	1,025	0.3	1	–	–	–	Insufficient RNA quantity for sequencing
11	2	Burns	Excitatory neurons	2,948	0.1	4.9	–	–	–	Insufficient RNA quantity for sequencing
12	2	Sham	Excitatory neurons	1,820	0.1	1.7	–	–	–	Insufficient RNA quantity for sequencing
13	2	Sham	Excitatory neurons	1,708	0.1	1.1	–	–	–	Insufficient RNA quantity for sequencing
14	2	Sham	Excitatory neurons	–	–	–	–	–	–	Unexpected adverse event; early EOE
**15**	**3**	**Sham**	**Excitatory neurons**	**11,145**	**11.3**	**6.8**	**56,232,850**	**90.37%**	**62.58%**	
**16**	**3**	**Sham**	**Excitatory neurons**	**3,138**	**7.1**	**7.7**	**54,510,420**	**90.22%**	**62.64%**	
**17**	**3**	**Sham**	**Excitatory neurons**	**6,678**	**14.5**	**7.2**	**50,835,808**	**90.24%**	**59.69%**	
**18**	**3**	**Sham**	**Excitatory neurons**	**8,223**	**31.2**	**8.3**	**42,191,808**	**89.50%**	**61.40%**	
**19**	**3**	**Sham**	**Excitatory neurons**	**5,762**	**11**	**8.5**	**52,359,648**	**90.52%**	**59.14%**	
**20**	**3**	**Burns**	**Excitatory neurons**	**5,462**	**9.5**	**8.8**	**68,858,866**	**90.57%**	**59.66%**	
21	3	Burns	Excitatory neurons		2.7	1	–	–		Insufficient RNA quantity for sequencing
**22**	**3**	**Burns**	**Excitatory neurons**	**13,767**	**8.8**	**8.9**	**42,613,900**	**90.61%**	**62.85%**	
23	3	Burns	Excitatory neurons		0.3	1	–	–	–	Insufficient RNA quantity for sequencing
**24**	**3**	**Burns**	**Excitatory neurons**	**16,916**	**8.7**	**7.1**	**59,087,792**	**90.47%**	**59.74%**	
1	1	Sham	Inhibitory neurons	–	–	–	–	–	–	Unexpected adverse event; early EOE
**2**	**1**	**Burns**	**Inhibitory neurons**	**34,274**	**21.6**	**9.5**	**77,139,283**	**92.51%**	**66.83%**	
3	1	Burns	Inhibitory neurons	25,604	14.5	9.7	125,719,847	92.67%	62.18%	Removed from analysis due to skin ulceration
4	1	Sham	Inhibitory neurons	–	–	–	–	–	–	Unexpected adverse event; early EOE
5	1	Sham	Inhibitory neurons	47,327	0.0	1	–	–	–	Insufficient RNA quantity for sequencing
6	1	Burns	Inhibitory neurons	57,617	0.0	1	–	–	–	Insufficient RNA quantity for sequencing
**7**	**1**	**Burns**	**Inhibitory neurons**	**48,522**	**23.1**	**10**	**111,706,354**	**92.11%**	**64.29%**	
8	1	Sham	Inhibitory neurons	50,024	24.4	9.8	87,173,148	92.68%	63.47%	Removed from analysis due to library size difference
9	1	Sham	Inhibitory neurons	–	–	–	–	–	–	Unexpected adverse event; early EOE
**10**	**1**	**Burns**	**Inhibitory neurons**	**7,816**	**5.1**	**9.8**	**94,435,884**	**92.90%**	**59.61%**	
**11**	**2**	**Burns**	**Inhibitory neurons**	**73,901**	**1.6**	**9.6**	**67,409,179**	**92.50%**	**66.84%**	
**12**	**2**	**Sham**	**Inhibitory neurons**	**71,321**	**2.7**	**10**	**73,617,064**	**93.39%**	**71.27%**	
**13**	**2**	**Sham**	**Inhibitory neurons**	**47,578**	**1.9**	**10**	**64,785,461**	**93.03%**	**67.26%**	
14	2	Sham	Inhibitory neurons	–	–	–	–	–	–	Unexpected adverse event; early EOE
**15**	**3**	**Sham**	**Inhibitory neurons**	**50,630**	**69.7**	**7.8**	**55,620,837**	**90.44%**	**64.16%**	
**16**	**3**	**Sham**	**Inhibitory neurons**	**47,102**	**27.5**	**7.3**	**54,421,113**	**90.35%**	**59.77%**	
**17**	**3**	**Sham**	**Inhibitory neurons**	**69,972**	**163.2**	**8.3**	**85,905,036**	**90.50%**	**60.85%**	
**18**	**3**	**Sham**	**Inhibitory neurons**	**89,049**	**97.1**	**8.2**	**56,931,708**	**90.64%**	**61.47%**	
**19**	**3**	**Sham**	**Inhibitory neurons**	**99,075**	**116.1**	**8.6**	**63,884,676**	**90.27%**	**63.52%**	
**20**	**3**	**Burns**	**Inhibitory neurons**	**70,955**	**78.9**	**8.7**	**82,638,029**	**89.78%**	**63.93%**	
**21**	**3**	**Burns**	**Inhibitory neurons**	**79,614**	**55.7**	**8.2**	**62,534,811**	**90.18%**	**66.58%**	
**22**	**3**	**Burns**	**Inhibitory neurons**	**60,524**	**10.2**	**7.8**	**70,332,968**	**90.42%**	**64.34%**	
**23**	**3**	**Burns**	**Inhibitory neurons**	**63,609**	**55.9**	**8.4**	**65,245,336**	**90.34%**	**66.23%**	
**24**	**3**	**Burns**	**Inhibitory neurons**	**42,484**	**22.9**	**8.1**	**63,135,581**	**90.12%**	**67.97%**	
1	1	Sham	Astrocytes	–	–	–	–	–	–	Unexpected adverse event; early EOE
**2**	**1**	**Burns**	**Astrocytes**	**5,677**	**8.1**	**9.4**	**107,774,127**	**92.85%**	**58.27%**	
3	1	Burns	Astrocytes	20,087	5.7	9.1	105,688,178	92.46%	60.56%	Removed from analysis due to skin ulceration
4	1	Sham	Astrocytes	–	–	–	–	–	–	Unexpected adverse event; early EOE
5	1	Sham	Astrocytes	19,235	0.0	1	–	–	–	Insufficient RNA quantity for sequencing
6	1	Burns	Astrocytes	8,011	0.0	1	–	–	–	Insufficient RNA quantity for sequencing
**7**	**1**	**Burns**	**Astrocytes**	**16,175**	**5.9**	**9.5**	**88,283,841**	**92.95%**	**61.57%**	
**8**	**1**	**Sham**	**Astrocytes**	**16,678**	**2.3**	**10**	**85,871,118**	**93.08%**	**58.68%**	
9	1	Sham	Astrocytes	–	–	–	–	–	–	Unexpected adverse event; early EOE
**10**	**1**	**Burns**	**Astrocytes**	**20,061**	**5.3**	**8.7**	**104,814,955**	**92.48%**	**67.09%**	
**11**	**2**	**Burns**	**Astrocytes**	**35,525**	**3.6**	**6.8**	**62,417,561**	**93.27%**	**65.81%**	
**12**	**2**	**Sham**	**Astrocytes**	**8,102**	**1.8**	**10**	**57,595,154**	**93.31%**	**66.92%**	
**13**	**2**	**Sham**	**Astrocytes**	**6,329**	**2.4**	**10**	**43,306,001**	**92.82%**	**61.59%**	
14	2	Sham	Astrocytes	–	–	–	–	–	–	Unexpected adverse event; early EOE
**15**	**3**	**Sham**	**Astrocytes**	**66,606**	**68.7**	**7.7**	**75,038,517**	**90.75%**	**59.19%**	
**16**	**3**	**Sham**	**Astrocytes**	**69,068**	**53.7**	**7.4**	**66,271,474**	**90.44%**	**59.00%**	
**17**	**3**	**Sham**	**Astrocytes**	**72,049**	**72.3**	**7.7**	**86,881,890**	**90.82%**	**60.13%**	
**18**	**3**	**Sham**	**Astrocytes**	**78,143**	**103.6**	**8.1**	**69,867,888**	**90.58%**	**60.85%**	
**19**	**3**	**Sham**	**Astrocytes**	**57,169**	**63.9**	**8**	**49,536,550**	**89.85%**	**57.80%**	
**20**	**3**	**Burns**	**Astrocytes**	**82,956**	**95.6**	**8.2**	**50,447,474**	**90.33%**	**58.65%**	
**21**	**3**	**Burns**	**Astrocytes**	**78,236**	**62.2**	**7.7**	**67,462,579**	**90.43%**	**63.61%**	
**22**	**3**	**Burns**	**Astrocytes**	**78,359**	**40.3**	**8.1**	**63,108,459**	**90.56%**	**59.99%**	
**23**	**3**	**Burns**	**Astrocytes**	**51,386**	**23.2**	**8.6**	**66,446,454**	**90.16%**	**61.64%**	
**24**	**3**	**Burns**	**Astrocytes**	**34,562**	**12.6**	**8.1**	**75,863,140**	**90.43%**	**65.17%**	
1	1	Sham	Microglia	–	–	–	–	–	–	Unexpected adverse event; early EOE
**2**	**1**	**Burns**	**Microglia**	**1,143**			**133,857,373**	**92.19%**	**56.24%**	
3	1	Burns	Microglia	2,612			140,242,605	92.60%	65.98%	Removed from analysis due to skin ulceration
4	1	Sham	Microglia	–	–	–	–	–	–	Unexpected adverse event; early EOE
5	1	Sham	Microglia	5,998			–	–	–	Insufficient RNA quantity for sequencing
6	1	Burns	Microglia	2,634			–	–	–	Insufficient RNA quantity for sequencing
**7**	**1**	**Burns**	**Microglia**	**2,246**			**130,672,576**	**92.84%**	**63.95%**	
**8**	**1**	**Sham**	**Microglia**	**1,736**			**147,610,456**	**93.14%**	**59.46%**	
9	1	Sham	Microglia	–	–	–	–	–	–	Unexpected adverse event; early EOE
**10**	**1**	**Burns**	**Microglia**	**3,689**			**115,722,149**	**92.65%**	**61.04%**	
11	2	Burns	Microglia	6,003			–	–	–	Insufficient RNA quantity for sequencing
12	2	Sham	Microglia	1,571			–	–	–	Insufficient RNA quantity for sequencing
**13**	**2**	**Sham**	**Microglia**	**1,613**			**59,642,784**	**93.49%**	**65.57%**	
14	2	Sham	Microglia	–	–	–	–	–	–	Unexpected adverse event; early EOE
**15**	**3**	**Sham**	**Microglia**	**10,231**	**54.9**	**8.5**	**61,786,059**	**90.35%**	**61.48%**	
**16**	**3**	**Sham**	**Microglia**	**10,600**	**77.7**	**6.8**	**66,271,474**	**90.64%**	**57.97%**	
**17**	**3**	**Sham**	**Microglia**	**13,197**	**52.2**	**8.2**	**64,277,776**	**90.51%**	**58.28%**	
**18**	**3**	**Sham**	**Microglia**	**18,091**	**60.8**	**8.4**	**61,764,666**	**90.66%**	**58.66%**	
**19**	**3**	**Sham**	**Microglia**	**10,930**	**53**	**8.4**	**72,201,490**	**89.87%**	**62.20%**	
**20**	**3**	**Burns**	**Microglia**	**10,785**	**70.2**	**8.5**	**64,434,525**	**90.64%**	**61.97%**	
**21**	**3**	**Burns**	**Microglia**	**8,719**	**24.8**	**8.4**	**63,205,943**	**89.87%**	**61.06%**	
**22**	**3**	**Burns**	**Microglia**	**9,476**	**34.1**	**8.4**	**55,479,355**	**90.17%**	**64.66%**	
**23**	**3**	**Burns**	**Microglia**	**15,130**	**29.6**	**8.7**	**86,224,268**	**90.58%**	**63.17%**	
**24**	**3**	**Burns**	**Microglia**	**5,958**	**14.9**	**9.4**	**71,382,787**	**90.62%**	**61.84%**	
